# Investigator and participant expectations for returning non-genetic results: insights from the Rare and Atypical Diabetes Network (RADIANT) study

**DOI:** 10.1017/cts.2023.684

**Published:** 2023-11-22

**Authors:** Forough Noohi, Manu S. Sundaresan, Rochelle N. Naylor, Lainie Friedman Ross

**Affiliations:** 1 Department of Medicine, University of Chicago, Chicago, IL, USA; 2 Department of Pediatrics, University of Chicago, Chicago, IL, USA; 3 Department of Health Humanities and Bioethics, University of Rochester School of Medicine and Dentistry, Rochester, NY, USA

**Keywords:** Atypical diabetes, monogenic diabetes, return of results, therapeutic misconception, nongenetic information, investigator-clinician duality, participant-patient duality, genetic exceptionalism

## Abstract

**Objectives/Goals:**

The Rare and Atypical DIAbetes NeTwork (RADIANT) aims to discover the underlying pathoetiology of atypical diabetes by conducting both genotyping and non-genetic deep phenotyping. While the return of genetic test results in research settings has been investigated, the return of non-genetic results (RoR-NG) has received less attention. We explore the RoR-NG with RADIANT investigators and participants.

**Methods/Study Population:**

We conducted one-on-one interviews with 10 adult RADIANT participants and 10 RADIANT investigators. Participants also completed two health literacy screening tools and a survey on perspectives regarding return of results (RoR). Investigators completed one survey on experience and confidence in explaining clinical tests utilized in the RADIANT study and another survey on perspectives regarding RoR.

**Results:**

Most participants were non-Hispanic White. All participants had high scores on health literacy screens. Both RADIANT participants and investigators expressed strong support for RoR-NG. RADIANT participants and investigators acknowledged the different roles and responsibilities between research and clinical care for interpreting and acting on non-genetic results. However, the lines between clinical care and research in returning and acting on results were often blurred by both participants and investigators.

**Discussion/Significance:**

Our study provides important insight into how both investigators and participants simultaneously distinguish and blur clinical and research roles and responsibilities when discussing non-genetic research results and the return of these results. Further study should engage individuals from diverse racial and ethnic backgrounds and with varying levels of health literacy to understand how best to support all participants when returning research results.

## Introduction

While the majority of diabetes can be classified as type 1 or type 2, there are numerous individuals with atypical forms of diabetes [1], for whom treatment approaches may be suboptimal [2]. The Rare and Atypical DIAbetes NeTwork (RADIANT) is a network of academic health centers across the United States that aims to discover and define rare and atypical forms of diabetes [3]. RADIANT participants proceed through up to three study stages of data collection with eligibility re-determined at each stage based on likelihood that the participant’s diabetes is both atypical and novel as described elsewhere [4]. Genetic and non-genetic data (laboratory, clinical phenotyping, and questionnaires), and omics data are collected in different stages (Table 1). RADIANT has policies about which results to return to participants, whether participants can opt in or opt out of receiving results and/or of sharing results with their clinical healthcare providers (HCPs), who may be primary care physicians, endocrinologists, or both. These RoR policies were developed by a consensus process of study investigators and a steering committee.

**Table 1.** Rare and Atypical DIAbetes NeTwork (RADIANT) data collection

**(A) Laboratory studies: Blood and urine**	**(C) Clinical data**
- Islet Autoantibody Testing	- Participant questionnaires
- Hemoglobin A1c	- Treating provider survey
- Lipids	- Medical record collection
- Comprehensive metabolic panel	- Medication Inventory
- Blood and urine collection for biobank storage	- Pedigree Collection
- Peripheral blood mononuclear cells collection	**(D) Omics data collection**
- Beta human chorionic gonadotropin	- Whole Genome Sequencing
	- Mitochondrial Genome Sequencing
**(B) Physical examination and clinical phenotyping**	- RNA sequencing (RNAseq)
- Anthropometrics	- Metabolomics
- Michigan Neuropathy Screening Instrument physical and self-assessment	**(E) Behavioral & other questionnaires**
- Hirsutism exam	- Automated Self-Administered 24-Hour (ASA24) Food Recall
- Tanner Staging	- Physical Activity and Physical Fitness questionnaire
- Acanthosis Staging	- Patient-Reported Outcomes Measurement Information System Cognitive Function and Global Health questionnaires
- Vision/hearing assessment	- Education Level Questionnaire
- Mini-Mental State Examination	- Environmental Exposure Questionnaire
- Photography	
- Magnetic resonance imaging (MRI)	
- Bone density scan (DEXA or dual X-ray absorptiometry)	

Participants can elect to receive diabetes autoantibody results in stage 1, genomic information obtained in a Clinical Laboratory Improvement Amendments (CLIA) certified laboratory in stage 2, and physical findings, laboratory, and cognitive test results in stage 3. Participants are not offered DNA variants of unknown significance and non-CLIA RNA sequencing data. Participants cannot opt out of receiving stage 3 urgent laboratory results (results falling below or above cutoff values established by the RADIANT investigators). Participant results are populated in the participant portal unless they decline the RoR. For urgent results that they cannot opt out of receiving, participants are notified via phone call and email. Participants choose whether to share stage 2 and/or stage 3 (non-urgent and urgent) results with their HCPs. Whether or not they go through all three stages, they are asked whether they would be willing to participate in other related studies (such as this one).

While many studies have explored expectations and impact of the return of genetic results (RoR-G), the return of *non-genetic test results* (RoR-NG) has received considerably less attention despite more overlap with routine clinical care. The conflation of research with clinical care is known as the therapeutic misconception [5], and while this misconception is often attributed to research participants, it can apply to the study investigators themselves [6,7,8]. In this mixed-method study, we explore participant and investigator perspectives about the RoR-NG. For patient-participants, we examined their decisions regarding the RoR and shared the information with their HCPs. We also asked patient-participants who should explain tests and test results, and who should order and pay for additional evaluation arising from abnormal research results. We queried RADIANT investigators about their perceived obligations to (1) return clinical and laboratory findings to research participants; (2) interpret and follow-up abnormal results; and (3) allow participants to abstain from receiving non-urgent abnormal results.

## Materials and Methods

### RADIANT Participants

We recruited adult patient-participants who agreed to the stage 1 consent form to be contacted for future research studies. Participants completed two measures of health literacy- the Rapid Estimate of Adult Literacy in Medicine (REALM) survey and the BRIEF health literacy screening tool [9,10]. Both surveys were conducted in less than ten minutes in total. Participants then took part in an interview (described below) that focused on their understanding of RoR to themselves and/or to their HCPs, and the responsibilities they believed the RADIANT investigators had with respect to these findings compared to their HCPs. Lastly, participants were asked to state their agreement (1 = least, 10 = most) with 5 statements focused on RoR and to provide some demographic data.

### RADIANT Investigators

Twelve of thirteen primary investigators who were trained to conduct the RADIANT stage 3 protocol were invited to participate (excluding RN who is a co-investigator on the project). Questions focused on their perspectives on sharing, explaining, and appropriate follow-up of research results as well as handling RoR in the clinical and research settings. Next, investigators completed a survey that asked about the frequency of ordering (never, rarely, sometimes, and frequent) and comfort with explaining results (10-point Likert scale) of seven lab tests (triglycerides, creatinine, estimated glomerular filtration rate, potassium, calcium, alanine aminotransferase, and hemoglobin A1c (HbA1c)) and two imaging procedures (abdominal MRI and DEXA) commonly used in routine clinical care and also included as components of the RADIANT study. The survey also asked about (1) experience with and (2) comfort in conducting a deep phenotyping physical exam and investigator demographics. Although the PHQ-8 and GAD-7 questionnaires (evaluating depression and anxiety, respectively) were eventually dropped from the RADIANT protocol, we asked about these surveys to explore investigator attitude about mental health responsibilities in the research setting. Lastly, investigators were asked to respond to ten statements regarding attitudes about the return of results (five-point Likert scale).

### Qualitative Structured Interviews

All interviews were conducted by F.N. in English. A consent document was sent by email prior to the interview. Oral consent, including permission for audio recording, was obtained. Interviews were conducted through video conference between April 2022 and September 2022 and were transcribed verbatim using Trint [11] (an audio transcription software), deidentified, and checked for accuracy by F.N. and M.S. The coding tree was developed by F.N., M.S., and L.R.

We used a thematic analysis to identify and analyze the data [12]. The codes were generated from the interview guide, and we inductively analyzed the data using ATLAS.ti (Version 22.0.2 (3332)) [13]. Saturation was reached within the first five transcripts for both participants and investigators. Further interviews were performed to establish the significance of both cohorts’ experiences and perceptions [14]. After coding was completed, codes with thematic similarities were merged [15,16].

### Human Subjects Protections

This is a supplemental study of RADIANT (approved by a central institutional review board (CIRB19-1488 and CIRB21-0710)). The supplement was reviewed by the University of Chicago Institutional Review Board as a minimal-risk study with waiver of written informed consent (IRB21-1411).

## Results

### RADIANT Participants

At the time of our study, twenty-one participants had completed all three RADIANT stages and had agreed to recontact. All were invited to participate and were interviewed on a first-come basis, although after seven interviews, we sent targeted reminders to male participants to get an even number of male and female participants. Ten participant interviews were conducted, each lasting between 60-100 minutes. All participants agreed to receive all eligible results from all stages of the study, and nine of ten chose to have their results sent directly to their HCPs. Only one had received an urgent result.

The demographics of the patient-participants are provided in Table 2. All participants were of high socioeconomic status (SES) and eight participants had post-secondary education. All participants scored 9 or 10 out of 10 on the REALM and 17–20 out of 20 on BRIEF, indicating adequate health literacy. Five statements about attitudes regarding the RoR found consensus (range 7–10) that results pertaining to medical conditions other than diabetes are helpful. All participants strongly disagreed (range 1–2) with the statement that results about medical conditions other than diabetes should not be shared. Participants were divided about whether results should be shared only if the investigators explained the results and whether the results should *always* be shared with clinical HCPs. Although no one strongly believed that the researchers had an obligation to help them get needed care if an abnormality was detected, a few believed there was some degree of obligation (See Table 3).

**Table 2.** Participants’ demographic information

ID (P#)	Biolo-gical sex	Age, decades	Race/ Ethnicity	No. of years since diabetes diagnosis	Educational attainment	BRIEF (maximum of 20)	REALM (maximum of 66)	Work status	Medical payment method
P1	F	70s	NHW	5	Some college	17	66	Retired	PI/Medicare
P3	F	30s	NHW	11	Graduate degree	20	66	Employed	PI
P4	F	60s	NHW	2	Graduate degree	20	66	Retired	Medicare
P7	F	50s	NHW	6	Graduate degree	18	66	Retired	PI, OOP
P8	F	30s	Asian	1	Graduate degree	20	66	Not employed-looking for work	PI, OOP
P2	M	50s	Black	9	Bachelor degree	17	66	Employed	PI, OOP
P5	M	60s	NHW	11	Graduate degree	20	66	Employed	PI
P6	M	50s	NHW	13	Bachelor degree	19	66	Not employed	Wife’s PI, OOP
P9	M	40s	HW	20	Some college	19	65	Employed	PI
P10	M	50s	NHW	<1	Graduate degree	20	66	Retired	PI, OOP

F = female; HW = hispanic white; M = male; NHW = non-hispanic white; P# = patient-participant number, OOP = out of pocket; PI = private insurance.

**Table 3.** Participant attitude about the RoR and the investigators’ responsibilities, ordered by strength of agreement (Ranking 1 = strongly disagree, 10 = strongly agree)

Statement order in survey	Statement	Mean ± standard deviation (range)
1	Results from this research study that pertain to medical conditions other than diabetes are helpful.	9.3 ± 1.1 (7–10)
4	Research results should always be shared with my health care provider.	6.6 ± 3.3 (1–10)
3	Research results should only be shared if the research investigator explains what they mean.	4.4 ± 2.9 (1–10)
5	If the researcher finds an abnormality, they have an obligation to help me get the needed clinical care.	4.3 ± 2.1 (1–7)
2	Research results about medical conditions other than diabetes should not be shared because I did not sign up for things unrelated to diabetes.	1.1. ± 0.3 (1–2)

### RADIANT Investigators

Ten of the twelve eligible RADIANT investigators participated in the interviews, which lasted approximately 60 minutes. Five investigators spent 50% or more of their time in clinical research. Ten investigators were asked 35 survey questions each, with only 11/350 (3.1%) missing data points. Eight investigators stated that they ordered the blood tests collected in the RADIANT study and DEXA scans either frequently or sometimes in clinical practice while only 60% frequently or sometimes ordered an abdominal MRI. All investigators rated their confidence at 7 or higher for interpreting all tests except the abdominal MRI, where only 70% rated their interpretation confidence at 7 or higher. Aspects of deep phenotyping were less frequently performed by respondents in clinical practice. While 100% frequently or sometimes performed thyroid examination and measured blood pressure, only half routinely performed a fundoscopic exam, examined patients for swollen axillary lymph nodes and even fewer frequently or sometimes performed cognitive testing or used the PHQ-8 or GAD-7 in clinical practice. Their confidence paralleled the frequency with which they performed these tests with 100% rating their confidence at 7 or higher for thyroid examination and measuring blood pressure but only 50% rating their confidence as high for the PHQ-8 and GAD-7.

Investigator agreement and disagreement with 10 statements about the RoR are reported in Table 4. All supported RoR to participants even if the participants did not have an HCP or insurance. Greatest differences of opinions were expressed about whether (1) participants should have the right not to have results shared with their HCP; (2) investigators have a responsibility to explain all returned results; and (3) clinically actionable results must be given to participants.

**Table 4.** Investigator attitudes about the return of results (arranged by % agreement)

Statement order in survey	Statement	Mean score	(% strongly or moderately agree)
8	It is the responsibility of the participant and their HCP to act (or not) on research results, even if I disagree with their course of action.	1.8	90
1	Participants should have the right to refuse all results.	2.0	70
6	As an investigator, I have a responsibility to return all results.	2.2	70
7	As an investigator, I have a responsibility to explain all returned results.	2.4	60
3	Participants should have the right not to disclose their results to their HCP and not to have results shared with their HCP.	2.7	60
2	Participants should NOT have the right to refuse clinically actionable results.	3.3	30
5	Abnormal results should be shared with the participants’ HCP regardless of whether the participants want the information to be shared.	4.1	20
4	Results should only be shared with the participant if the participant has a sufficient level of health literacy to understand the results and or what to do with them.	4.5	10
9	Research results should not be shared if the participant does not have access to an HCP.	4.5	0
10	All research participants should be required to have health insurance in case of an abnormal research finding or an abnormal reaction to a research intervention.	4.8	0

HCP = health care provider.

Scoring: (1 = strongly agree; 2 = moderately agree; 3 = neutral; 4 = moderately disagree; 5 = disagree).

The interviews identified 10 primary themes (7 for participants and 3 for investigators) (see Table 5).

**Table 5.** One-on-one interviews, themes, and sub-themes

Participants
Diagnosis of Atypical Diabetes
Experience with accessing and understanding electronic health records
Motivations for Participating in RADIANT
Comprehension of the consent forms and the return of results
Receiving general research results
Participants’ choices on receiving and sharing results
Communication of abnormal and urgent results
Physical exam (clinical routine vs. RADIANT)
Responsibility: the line between research and clinical practice
Investigators
Responsibility: the line between research and clinical practice
The notion of actionable results
Financial and incidental barriers

*Quotes from RADIANT participants (De-identified quotes are labeled by participant number, followed by F (Female) or M (Male) and age in decades.)*

### Diagnosis of Atypical Diabetes

Getting an atypical diabetes diagnosis is often not straightforward. Some participants explained that they were described as atypical from symptom-onset because they had an unexpected phenotype:

You know, my doctor, you know, expressed surprise. Because my body type is not what they typically think… So there wasn't much more than that other than “How do we manage it?” [P7, F, 50s]

Others were diagnosed with type 2 diabetes and then had an episode of DKA challenging their initial diagnosis.

… just before I turned 60 […] my A1C turned up 6.1, and at that point, the doctor diagnosed me as type 2… And so, another few years down the road was when I had the DKA at 64. [P4, F, 60s]

So, I was first diagnosed about ten years ago, at this point. I didn't really have any signs or symptoms really, but, you know, just kind of had some routine blood work and was told that I was diabetic…[In] October of 2020. So, I was actually hospitalized for a couple of days because I, basically I had gone into diabetic ketoacidosis. And so, kind of as a result of that, changing my treatment plan and then sort of having a meeting with my endocrinologist. [P3, F, 30s]

### Experience With Accessing and Understanding Electronic Health Records

All participants reported access to their clinical lab results, imaging, and chart notes through an electronic health record portal. In RADIANT, research results are also returned through an electronic portal. While most participants found the RoR in the clinical setting similar to the RoR in the RADIANT portal, others noted differences. Notably, participants stated that clinical results were returned more quickly and provided more interpretation and/or opportunities to discuss the results with the health care team.

Well. The clinical results come usually more quickly. And there’s a follow-up within a week or so to discuss them. [P4, F, 60s]

Um, I guess there weren't, there weren't notes. I mean, like, when I see my primary, I'll get notes summarizing, and there were no notes. It was just the data and the results. [P7, F, 50s]

### Motivations for Participating in RADIANT

While participants came to the study by varying methods (referral by HCPs, referral from RADIANT sites, and self-referral following online searching or social media advertisements), all participants hoped to receive information that would explain why they developed diabetes or help to classify their diabetes type.

Yeah. I want to get to the bottom of my diabetes and see what I have because it doesn't fall into any category. So that’s what I wanted, was just to get some answers on what I have. [P8, F, 30s]

For me, it’s to hopefully understand better the cause for my glucose intolerance, my diabetes, if there are better methods to treat it. And how common it is. [P1, F, 70s]

Many participants acknowledged that the RADIANT study might not provide an immediate explanation for their atypical diabetes but were keen to participate to advance science:

One of my endocrinologists, actually, they could not explain how this had developed so rapidly. They just said, would you be interested in, and they explained that it might not explain mine, or that it’s just background research information. And I said, sure, why not? [P10, M, 50s]

Well, this study, the objective I hope to gain wouldn't benefit myself. It’s really just to kind of, the information that can be gathered and utilized for future and for anyone else that might have the same type of symptoms that I might have. [P2, M, 50s]

Several also clearly stated that one of their aims was to help future generations—either their own children or others similarly situated:

You know, a better understanding of why people like me get diabetes? I do not know that I will have the understanding, necessarily, but the medical community, to hopefully help my son or my grandkids get it. So it’s more proactive. [P7, F, 50s]

As time went on, I realized that I was feeling very positive about something good coming out of something so bewildering and kind of scary. And that would be to help someone else. Down the line, someone I will probably never know or meet. [P4, F, 60s]

### Comprehension of the Consent Forms and The Return of Results

All participants had either read the consent forms (for all RADIANT stages) and/or had the consent forms read to them. All participants indicated that they had understood the content of the consent forms and the policies on the return of results and that their questions were answered by the study staff:

[…] I guess for all three, the option to share the information with a provider, whether I wanted to do that or not. And for stage two and you know, specifically, the information about what I could choose to receive in terms of the genetic testing, whether it would be limited to just kind of looking for the diabetic markers, whether I wanted that or and also included like, you know, just kind of outside of that other markers and whether I wouldn't want to receive any of that information and again, whether I would want that shared with a provider. [P3, F, 30s]

### Receiving General Research Results

#### Participants’ choices on receiving and sharing results

As mentioned, all participants agreed to receive all results from all stages of the study, and all agreed to have their results sent directly to their HCP (i.e., primary care providers and/or endocrinologists), except for one participant who had concerns regarding privacy issues:

I wanted the results to come to me. And then I could choose on my own, outside of the study, to give results to my physician, show results to my physicians […] the study is potentially collecting my genome information…And, you know, you could be denied insured health insurance or life insurance or things like that. So, I wanted to know what the protections were, were there. [P5, M, 60s]

#### Communication of abnormal and urgent results

Results were available in the participant portal. Other than diabetes indicators which would be expected to be abnormal, only one participant received an abnormal urgent result from the RADIANT study to date, although that turned out to be a spurious result:

So, one of my results came back kind of sky high, through the roof…I received a call from [the doctor] letting me know, like asking if I was okay and just letting me know of the high results and, you know, again, asking if I could, you know, go ahead and get my labs done again, just to make sure everything was okay and that it was just an error. [P3, F, 30s]

### Physical Exam (Clinical Routine vs. RADIANT)

The stage 3 visit involves a detailed physical exam and numerous anthropometric measurements and photographs for documenting physical appearance to look for disease manifestations that could help explain an individual’s atypical diabetes. All participants stated that the physical exam performed by the RADIANT investigators was different or at least substantially more thorough than what was performed in their usual checkup:

…I try to think nobody bangs my knee to see how my reflexes are. Do you know what I mean? It was just a whole different experience. Clearly, it was about getting information about a physical body’s situation. Didn't necessarily have to do with spotting problems. It’s about gathering information in order to have a full picture of a study subject. [P4, F, 60s]

The RADIANT manual of procedures does not specify communication of physical exam findings, although most of the investigators said they would share findings that required interventions. Some of the participants described being reassured that their feet were healthy or that they had good balance but most stated they were not given specific results and that they did not know what the investigators were looking for:

…stage three was mostly a physical exam. They did all kinds of weird stuff! They poked and prodded and photographed, their balance test and measured the dimensions of my face. I have no idea what they were looking for there. [P5, M, 60s]

### Responsibility: The Line Between Research and Clinical Practice

Most participants stated that they recognized the difference between the role and responsibilities of their treating physician compared to RADIANT researchers’ responsibilities in following up with their abnormal results. Still, several participants believed that RADIANT researchers had an obligation to provide the necessary information obtained through research to facilitate their clinical care.

I think there’s an obligation to make sure that the person [has] the opportunity to know that information and to provide ways of giving clinical care […]. You know, I do not think it’s really the study’s responsibility to follow up and make sure that they actually are doing that. But I think it’s the responsibility of notifying, providing some guidance and helping to coordinate some of that. [P3, F, 30s]

Well, in my case, it’s the same person doing both. [The RADIANT investigator] is my physician, he’s supposed to be looking out for my best interests and trying to think about how to manage my diabetes. In his role in RADIANT, he’s trying to think more broadly and try and understand or just help discover what causes various atypical, causes of atypical diabetes might be. And, you know, if those findings do not have a direct relevance to my health care, I don't think he’s obligated to share them with me. [P5, M, 60s]

### Quotes From RADIANT Investigators

#### Responsibility: the line between research and clinical practice

Investigators expressed conflicting feelings when discussing that participants might not want to know their abnormal results or that they might not want their treating physicians to learn about their participation in research and/or about the research results. Many of our investigators wanted to re-confirm the refusal and/or sought out workarounds:

But I think we do have an obligation. Again, I mean, as, as physicians. But even I would say as, as researchers, if there’s something that has a potential like real health implications that we do, we need to do our best to inform them and let them know. [INV.7]

Despite misgivings, however, investigators expressed that they would respect participants’ autonomy to refuse the return of abnormal, non-urgent results and/or to refuse to share the results with their HCPs. Some investigators justified respecting participant refusal of RoR-NG and not following up on abnormal results by distinguishing between their responsibility as clinicians versus their responsibility as investigators:

I mean, I think that the responsibility is to convey the information and advise them to follow up with their provider regarding it, I think. I don't think that I need to call the person the following week and make sure they did it. [INV.3]

No, I wouldn't [follow up], because like I said, that is now the patient’s responsibility. My responsibility was to inform them that they need to go see their specialist or their primary care, but then they have to take ownership of their health after that. [INV.10]

Others, however, expressed a discomfort in distinguishing between their dual roles:

Oh, I think my responsibility is the same because I owe that information to the patient. So, the responsibility is definitely the same. And if there was an abnormal lab, I would try to communicate the same way I would do to a clinic patient [INV 10]

Well, I try. I mean, it turns out that sometimes there’s, it’s a blend. There are times when it’s impossible to separate and, and it has to do with the nature of the research. In clinical research I think inevitably, although maybe people would argue with me, there are findings that happen in research, which, which are actionable, which affect the quality of life for the diagnosis and some other intervention, the patient. And so you have to be able to cross that line or two to make sure that the patient’s physician understands the ramifications. So, I think I mean, to take it, I think there’s a moral imperative to be sure that the results of the investigation are communicated. So, I, I do try to figure out how to straddle that area. [INV.1]

#### The notion of actionable results

The most notable difficulty investigators had in distinguishing between research and clinical care was whether non-urgent but abnormal results were “actionable.” Investigators agreed in defining this as a result that should or could result in changes in diagnosis or treatment, but some did not feel that *urgent* results as defined by the RADIANT protocol would necessarily capture all *actionable* results. There was additional confusion as to what action might be appropriate for a RADIANT investigator to take.

Let’s say somebody’s potassium comes back low. And I know they're on a diuretic…if somebody’s calcium comes back at 2.9, which is low, yes, that’s an actionable item [defined as urgent in RADIANT]. But to me, the bigger question is, somebody’s potassium comes back at 2.9, and it’s part of RADIANT. Then who, who’s medically, legally responsible for that? Because I'm not the one prescribing the diuretic. So, do I call the patient, or do I call the primary doctor? [INV.8]

Additionally, those who did feel that urgent and actionable results were synonymous in the RADIANT study still noted that actionability being defined in the protocol removed the ability to use clinical judgment and personalize their response.

I mean, in practice, I think there’s a lot of clinical judgment that goes into what’s an actionable item in practice. But in most of the research protocols that I've been involved with, we define what’s actionable as part of the protocol. [INV.3]

#### Financial and incidental barriers

All investigators believed that insurance should not be a requirement to participate in research as that would increase inequities, while concurrently acknowledging the difficulties it would cause with following up on incidental findings:

I mean, the possibility of secondary findings is not a reason to exclude them. In fact, it’s a reason to… it’s a good reason to bring them in, because, frankly, we have a lot of patients who only get their primary care through participating in one of our studies, you know. [INV.6]

I think to make a requirement [for health insurance] would be more of a hindrance than a benefit because I think you would be self-selecting a certain group of people. […] and if one of our goals is to actually recruit underserved populations or racial minorities, we would, we would be missing a lot. No, I think it does open a can of worms in saying, you know, follow up with a medical provider. [INV.3]

## Discussion

Little attention has been paid toward the attitudes of clinician-researchers and participant-patients surrounding the RoR-NG. Our study provides important insight into two different debates in the literature: 1) the significance of RoR-NG; and 2) the distinctions between patient and research participant, clinician and investigator, and patient-clinician versus participant-investigator relationships.

### Return of Results

While clinicians and researchers were historically hesitant about RoR, in the past two decades there has been a shift to acknowledge the value of the participant’s role in the research study and greater willingness to share results—even when their meaning is not fully understood [17,18]. Given the strong personal curiosity and motivation shown by the participants, it is not surprising that all participants consented to the return of all eligible genetic and non-genetic results. Many participants described how they would research and/or discuss the findings with their HCPs to interpret the results.

Similarly, all investigators supported RoR-G and RoR-NG within the limits set up by the RADIANT protocol even if participants lacked access to providers who could help them use the information clinically. With respect to RoR-NG, our investigators wanted to return non-urgent, but still clinically actionable results even if the participant did not want to receive them, reflecting the tension between their instinct to act in what they deemed to be the participant’s clinical best interest versus their role as objective scientists. The blurring of their obligations to patients versus to participants is further explored below.

### Blurring the Line Between Clinical Care and Research

Both our qualitative and quantitative data showed a blurring of lines between clinical care and research. Participants expected their participation to both advance science and provide personal health insights. While they acknowledged a difference between the RADIANT investigators and their own clinicians, there were some who felt that the investigators had “the responsibility of notifying, providing some guidance and helping to coordinate some of that [follow-up care for abnormal results].” In the interviews. investigators stated that they would (begrudgingly) respect participants’ autonomy and not force participants to receive their abnormal non-urgent results. However, RADIANT investigator survey results showed 30% moderately or strongly agreed that “participants should not have the right to refuse clinically actionable results,” and 20% felt that “abnormal results should be shared with the participant’s HCP regardless of whether the participants want the information to be shared.”

Both patient-participants and investigators acknowledged the boundaries of their respective roles. The patient-participants realized that there were limits to the researchers’ obligations to help participants get needed clinical care if abnormalities were discovered, but they expected the investigators to make sure they were aware of them. The investigators also perceived differences, acknowledging being more proactive about checking results in the clinical versus research setting and being more directive about how to proceed when results are abnormal in the clinical setting. The investigators also expected participants to take greater ownership of their research results, and to explore next steps with their own HCPs if the results required follow-up. Yet, the investigators felt uneasy ignoring abnormal test results, and many would go out of their way to ensure that the participant followed up on the results.

The differences between clinician and investigator responsibilities were deliberated upon when the RADIANT investigators debated whether to exclude the GAD-7 and PHQ-8 questionnaires from data collection. The reason not to prospectively collect these data was concern about investigator responsibility to a participant with acute mental health challenges. While all RADIANT investigators discussed taking clinical responsibility for a patient who expresses acute depression or acute anxiety (often by walking them to the nearest emergency department), they were more reticent to undertake this responsibility for a research participant with whom they had no previous relationship. During the final planning stages, there was consensus to avoid the significant disruption that an acute mental health finding might have for the research participant and the study more generally.

### Limitations

One limitation of our study was that it was conducted early in the RADIANT implementation stage and most investigators had not yet had to deal with returning urgent results. In fact, at the time our study was completed, only one urgent metabolic result had been returned, and on further testing, it was determined to be spurious. Returning abnormal cognitive results may create an even greater challenge that should be evaluated in future studies in which results are returned.

Second, while we discussed urgent and non-urgent results as defined by RADIANT with patient-participants, we explored the concept of actionability only with the investigators, as the nuances between these may be beyond the scope of a layperson. Thus, we cannot exclude additional “mismatch” between participants and investigators in this regard.

Third, many individuals with atypical diabetes are either not identified or misclassified. To identify a person as having atypical diabetes requires a high level of expertise on the part of the provider and often requires a high degree of health literacy and self-advocacy on the part of participants. A limitation of our data is that all our participants had high health literacy scores and high SES. Although not all participants within RADIANT are of high health literacy and high SES, the lack of diversity of participants is a problem of rare disease recruitment more generally [19,20,21] given the barriers to inclusion: the need to understand that their diabetes was not typical, the ability to invest time into advancing science even though they may not get a diagnosis themselves, and the time and flexibility to participate.

The RADIANT team is actively working to engage individuals from all SES and educational attainment status, with an intentional focus on those traditionally underrepresented in research to ensure that the knowledge benefits of this study accrue broadly. RADIANT utilizes visual recruitment ads that are accessible across varying health literacy levels and provide continuing medical education opportunities to primary care doctors in diverse practice settings to increase awareness and referral. Ascertaining perspectives on RoR-NG from individuals with diabetes from diverse racial and ethnic backgrounds and with varying levels of health literacy and SES and from the clinicians who care for them will be an important future direction for informing best practices for RoR to participants.
